# Transposon sequencing analysis of *Bradyrhizobium diazoefficiens* 110*spc4*

**DOI:** 10.1038/s41598-021-92534-z

**Published:** 2021-06-24

**Authors:** Claudine Baraquet, Weijun Dai, Jose Mendiola, Kieran Pechter, Caroline S. Harwood

**Affiliations:** 1 Laboratoire MAPIEM, Université de Toulon, Toulon, France; 2 Guangdong Province Key Laboratory of Microbial Signals and Disease Control, Integrative Microbiology Research Centre, South China Agricultural University, Guangzhou, 510642 China; 3 Department of Microbiology, University of Washington, Seattle, USA; 4 The Children’s Hospital of Philadelphia, Philadelphia, PA USA

**Keywords:** Bacteriology, Environmental microbiology

## Abstract

*Bradyrhizobium diazoefficiens* USDA110 is one of the most effective nitrogen-fixing symbionts of soybeans. Here we carried out a large-scale transposon insertion sequencing (Tn-seq) analysis of strain Bd110*spc4*, which is derived from USDA110, with the goal of increasing available resources for identifying genes crucial for the survival of this plant symbiont under diverse conditions. We prepared two transposon (Tn) insertion libraries of Bd110*spc4* with 155,042 unique Tn insertions when the libraries were combined, which is an average of one insertion every 58.7 bp of the reference USDA110 genome. Application of bioinformatic filtering steps to remove genes too small to be expected to have Tn insertions, resulted in a list of genes that were classified as putatively essential. Comparison of this gene set with genes putatively essential for the growth of the closely related alpha-proteobacterium, *Rhodopseudomonas palustris*, revealed a small set of five genes that may be collectively essential for closely related members of the family *Bradyrhizobiaceae.* This group includes bacteria with diverse lifestyles ranging from plant symbionts to animal-associated species to free-living species.

## Introduction

*Bradyrhizobium diazoefficiens*, formerly named *Bradyrhizobium japonicum*^1^, is an alpha-proteobacterium that infects soybeans (*Glycine max)* to form nitrogen-fixing root nodules that provide useable nitrogen to the host plant. With its outstanding nitrogen-fixing abilities^2–6^, *B. diazoefficiens* USDA110 has been established as a model organism in studies of the soybean-bacterial symbiosis. Although many genes important or required for symbiosis have been identified, many fewer genes have been identified that are important for the fitness of USDA110 or its derivatives in other environmental settings, such as drought-stressed soils or in soils near or distant from soybean roots.

A now standard method to identify bacterial genes that are important for viability under a particular condition is transposon (Tn) sequencing (Tn-seq)^7–11^. Tn-seq is based on the generation of a transposon insertion library of a bacterium of interest. Hundreds of thousands of Tn insertions can be identified simultaneously by high-throughput sequencing and those genes which receive few or no insertions are classified as putatively essential under the conditions being interrogated^12^. To provide a community resource that we or others can use to identify genes important for fitness, we generated a transposon insertion library of Bd110*spc4*, mapped the sites of Tn insertions, and applied bioinformatic analysis to estimate a set of putative essential genes required for aerobic growth on yeast extract- arabinose-gluconate (AG) medium. Bd110spc4 is a spontaneous spectinomycin resistant mutant of *B. diazoefficiens* USDA110^13^.

This baseline data set can be used in conjunction with the Bd110*spc4* transposon insertion library that we generated to identify genes that are needed for Bd110*spc4* growth or survival in natural conditions or in a specific condition in the laboratory. We found immediate utility for the list of Bd110*spc4* putative essential genes when we used multiple BLAST comparisons to identify a small set of five genes that we hypothesize to be collectively or conditionally essential for a subset of related species in the family *Bradyrhizobiaceae* with diverse lifestyles but not for other alpha-proteobacteria.

## Results

### Transposon library construction

Two Bd110*spc4* libraries were generated by delivering suicide plasmid pLG107^11^ from *Escherichia coli* strain SM10 $\mathrm{\lambda }$pir to Bd110*spc4*. pLG107 carries the Tn5-based transposon T24 (IS*lacZ*-p_rhaBo_/FRT-kan). Individual conjugative mating mixtures were plated on yeast extract-arabinose-gluconate (AG) agar supplemented with kanamycin (Km) to select for Tn insertions and chloramphenicol to select against the *E. coli* donor strain, since Bd110*spc4* is naturally resistant to chloramphenicol. Two mutant libraries were prepared, and the Tn insertions were mapped by Illumina sequencing to the USDA110 genome (BA000040)^6^ as described in Materials and Methods.

### Identification and analysis of putative essential genes

We pooled data from five sequenced samples from two Tn libraries (MP1 and MP2) that we prepared, leading to the identification of 155,042 unique Tn insertions, 129,275 of which are in predicted genes and 25,767 of which are located in intergenic regions (Table 1) (Tables S1–3). We carried out a series of computational steps to generate normalized read values for each of the 8317 annotated protein-encoding genes (Table S4). First, the number of reads were normalized to a total of 10^7^ reads per sample (see Table S1–3 in the supplemental material). Second, since we did not consider Tn insertions in the first 5% or the last 10% of each gene, the total number of reads per gene were normalized based on 85% of the gene length, resulting in a 5–90 RpK value. Log_2_ [5-90RpK normalized] values were then plotted against numbers of genes (See Figure S2 in Supplemental Material). To identify genes that were required for growth under the conditions of the Tn mutant library construction, we conducted a Gaussian distribution analysis to find genes with no or very low RpK values compared to the rest of the genes. For this we used a cut-off of 99.9% confidence interval based on log_2_5-90 RpK (Figure S2). Putatively essential genes were defined as those with RpK values below this cut-off for each of the five samples analyzed (Table S4). The predicted essential genes were further filtered to remove genes too small to be expected statistically (P-value < 0.005) to have an insertion based on the density of Tn insertions that we observed. This resulted in the removal of genes with a length < 310 bp from consideration as essential genes.

**Table 1:** Bd110*spc4* transposon library composition.

	Mutant pools^a^				
	Mp2-H1	MP2-H2	MP2-M1	MP1-A1	MP1-B1
Reads	46,099,343	49,847,590	6,308,445	55,312,045	13,533,545
Mapped reads	31,014,801	34,474,023	4,458,797	2,661,007	878,118
Unique insertion sites	67,580	71,578	66,235	55,303	47,917
Total unique insertions	155,042				
Genome size (bp)	9,105,828				
Insertion frequency (bp/insertion)	58.7				

^a^Technical replicates of Master mutant pool 2 (Mp2-H1, Mp2-H2 and MP2-M1) and Master mutant pool 1 (MP1-A1 and MP1-B1) were prepared and sequenced. Reads were mapped to the *B. diazoefficiens* strain USDA110 as a reference genome.

**Figure 1:**
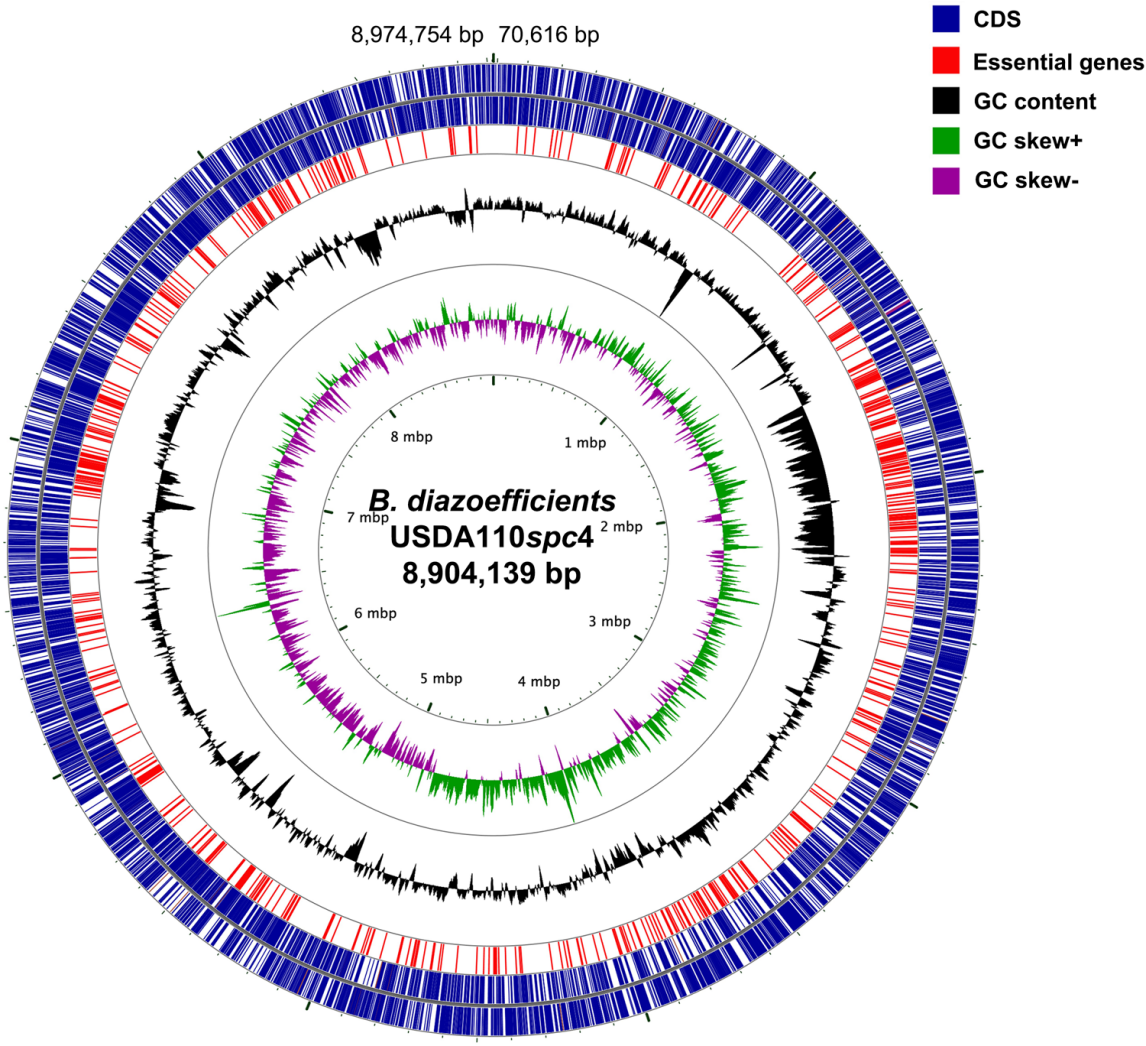
The chromosomal distribution of essential genes in *B. diazoefficiens* 110*spc4*. These were mapped to the USDA110 genome. The sequenced genome of *B. diazoefficiens* USDA110 was reported to be 9,105,828 bp^6^. In the figure, we have removed nucleotides spanning the region from 8,974,754 to 70,616 bp of the originally sequenced genome (as indicated by the numbering at the top of the figure), as we now know that these have been deleted from Bd110*spc4*^14^. For this reason, we have labeled the genome as USDA110*spc4.* From the outside in, the plus strand of CDS, the minus strand of CDS, location of essential genes, GC percentage and GC skew. Essential genes are shown in red.

From this analysis, we noticed that one region of the USDA110 genome of 9.1 Mb had almost no Tn insertions. Shortly after this, a report was published showing that Bd110*spc4* has a deletion of genes *blr0001*-*bsr0078* and *blr8173-bsl8317* encompassing a contiguous region of about 202 kb that includes 223 genes as well as a few other changes, resulting in a genome size of approximately 8.9 Mb^14^. Bd110*spc4*, which has been in laboratory culture since 1983^13^, has maintained its nitrogen-fixing abilities and is not defective in symbiosis^14^. In estimating the number of putatively essential genes, we used the USDA110 genome sequence and annotation, minus the 223 genes that have been deleted from the Bd110spc4 genome (Fig. 1), resulting in an estimate of 622 putative essential genes (Table S5). This is in the same range as the number of putative essential genes inventoried for other bacteria in other studies^8, 11, 15–19^.

**Figure 2:**
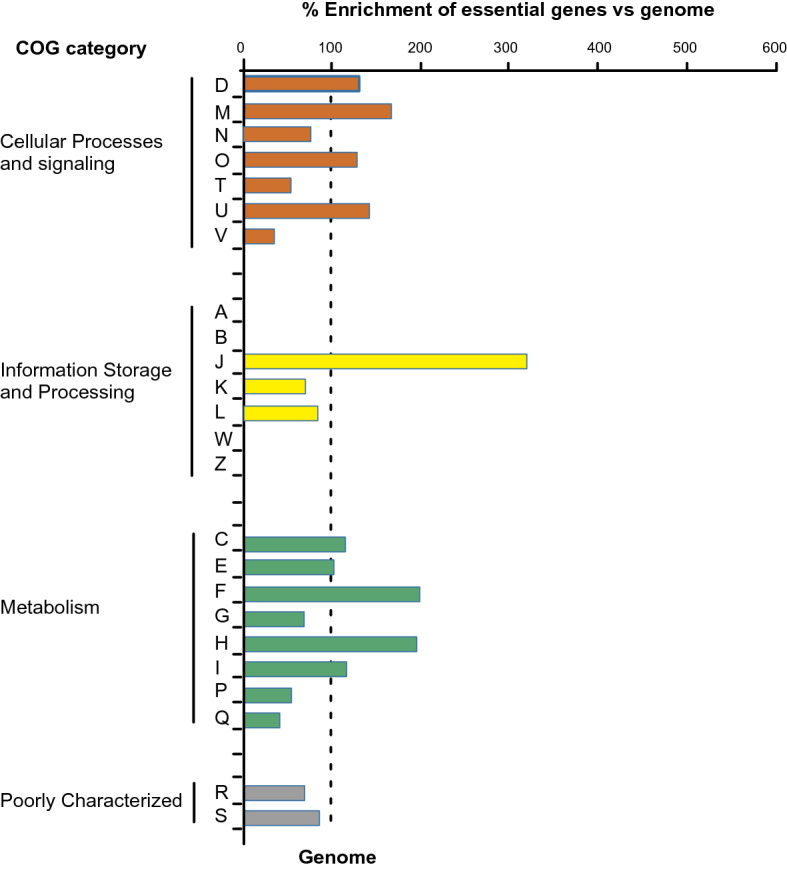
Functional class enrichment analysis of essential genes based on Cluster of Orthologous Groups (COG) categories. The subset of genomic genes contributing to each COG category was set to 100%. The percentage of essential genes within a COG category was calculated by comparison to genomic genes. D, cell cycle control, cell division, chromosome partitioning; M, cell wall/ membrane/envelope biogenesis; N, cell motility; O, posttranslational modification, protein turnover, chaperones; T, signal transduction mechanisms; U, intracellular trafficking, secretion, and vesicular transport; V, defense mechanisms; J, Translation, ribosomal structure and biogenesis; K, Transcription; L, Replication, recombination and repair; C, energy production and conversion; E, amino acid transport and metabolism; F, nucleotide transport and metabolism; G, carbohydrate transport and metabolism; H, coenzyme transport and metabolism; I, lipid transport and metabolism; P, inorganic ion transport and metabolism; Q, secondary metabolites biosynthesis, transport and catabolism; R, general function prediction only; S, function unknown.

The set of putative essential genes that we identified has a similar enrichment of functions to those reported for essential gene sets from other bacteria. Of the 622 protein-encoding genes identified, 511 genes were assigned a Clusters of Orthologous Groups of proteins (COG) identifier (Fig. 2) (Table S6)^20^. Relative to the numbers of genes in each category in the USDA110 genome, there was an enrichment of putative essential genes in categories D (Cell cycle control), F (Nucleotide transport and metabolism), H (Coenzyme transport and metabolism), M (Cell wall, membrane and envelope biogenesis), U (Intracellular trafficking, secretion, and vesicular transport) and over a three-fold enrichment in category J (Translation, ribosomal structure and biogenesis).

### Our data set has caveats

Having said this, we also know that the Tn library that we generated does not reflect the number of Bd110*spc4* genes that are truly essential with complete accuracy. Recent studies of other bacteria have generated datasets of hundreds of thousands of Tn insertions and this is accompanied by an increase in accuracy. For reasons described in the methods section, the generation of the Bd110*spc4* Tn libraries was laborious, and the total number of Tn insertions present in the Tn libraries that were generated were lower than we would have liked. However, in view of the fact that no other Tn-seq analysis of any *Bradyrhizobium* strain appears to have yet been reported, we decided to move forward with our libraries of 155,042 unique insertions. Our complete analysis can be found in the accompanying Supplementary Tables. In the following two paragraphs we describe caveats that are evident from our analysis of the Tn insertion data.

One is that our list of 622 putative essential genes, includes genes that we know not to be required for aerobic growth of any bacterium on a complex medium such as AG. These include some nitrogen fixation genes, hydrogenase genes, nodulation genes, conjugal transfer genes*, rchl* type 3 secretion genes, *cbbS*, encoding the small subunit of rubisco, flagella genes, and the regulator gene *regR*. One reason why some non-essential genes appear on putative essential gene lists is transposon Tn5 and its derivatives do not insert completely randomly into DNA. This is clear from looking at Tables S1 and S2, where one can see that Tn insertion points across genes are not evenly distributed and that the Tn can insert into the exact same site with high frequency. In general, Tn5 inserts into regions of high GC content much more efficiently compared to more AT rich regions^21^. In this regard it is interesting to note that most of the many putatively essential genes that we know to not to be needed for growth on AG medium, are located in the large 680-Kb symbiosis island that is located at genome coordinates 1681–2362 Kb (Table S5). The symbiosis island has a considerably lower GC content than the rest of the genome (Fig. 1). In all likelihood, the symbiosis island encompasses 123 false positive essential genes (shaded in yellow in Table S5).

A second caveat is that some genes that we expected to be essential were not identified as essential in Bd110*spc4*. Examples are RNA polymerase genes and some ribosomal proteins genes. In instances such as these, we found it important to look at the Tn insertion data carefully. Most of ribosomal protein genes identified as non-essential have Tn insertions located only at the beginning and/or the end of the gene, suggesting that only a segment of the gene is essential, but our library is not dense enough to allow highly accurate identification of the entire essential genome. We found that using a less stringent cut-off for essentiality revealed additional genes that may be important for cell viability (Table S4). For example, genes *bll5409* and *bll5410* encoding the beta and beta prime subunits of RNA-polymerase each met the criteria for essentiality in four of the five Tn library samples sequenced and are scored as reduced fitness genes in the fifth Tn library sample. Another example is succinate dehydrogenase, a four-subunit enzyme that is part of the tricarboxylic acid cycle. The four genes encoding this enzyme are essential in *R. palustris* but not in *E. coli* and one of them (*blr0515*) is putatively essential in Bd110*spc4*. Inspection of the data in Table S4 shows that the full set of succinate dehydrogenase genes is essential in three of the five Tn library samples analyzed. From this we conclude that succinate dehydrogenase probably is necessary for viability of Bd110*spc4* under the conditions in which we constructed the library.

### Genes that may be collectively essential for a subgroup of the Bradyrhizobiaceae

We were interested to see that five genes that scored as necessary for growth of both Bd110*spc4* and *R. palustris* CGA009 do not have homologs in *E. coli* or the alpha-proteobacterium *Caulobacter crescentus*^11^. A Blastp analysis showed that the top hits to these five Bd110*spc4* genes were to homologues present in species of *Afipia*, *Nitrobacter*, *Tardiphaga, Oligotropha* and *Rhodopseudomonas* (Table 2). These genera form a subgroup within the family *Bradyrhizobiaceae*^22^. We were interested to see that four of the five genes that we hypothesize to be essential for members of the subgroup of *Bradyrhizobiaceae* based on Tn-seq, encode hopanoid biosynthesis enzymes. Hopanoids have some of the properties of sterols. They comprise a small percentage of membrane lipid and confer rigidity to phospholipid bilayers and to the lipopolysaccharide layer of outer membranes^23, 24^. The basic cyclic triterpenoid hopanoid structure is derived from squalene by the squalene-hopene cyclase (encoded by *sqhC*) in an oxygen-independent reaction. Three genes, *hpnC*, *hpnD* and *hpnE* catalyze the biosynthesis of squalene. Squalene synthase, encoded by *hpnC*, is considered to be the first committed step in hopanoid biosynthesis^25, 26^. In past work investigators attempted but failed to isolate a *sqhC* mutant of *B. diazoefficiens*, which supports our data presented here showing hopanoid synthesis is required for growth^27^. A *sqhC* deletion mutant of a different species, Bradyrhizobium BTAi1, has been described that is defective in growth and sensitive to a variety of stresses^24^.

**Table 2:** Genes deemed essential in Bd110*spc4* that are common to a subgroup of the *Bradyrhizobiaceae.*

Locus tag	Gene name	Annotation	E value^a^
blr7762	-	homospermidine synthase	0
blr3004	*sqhC*	squalene-hopene cyclase	0
blr3003	*hpnE*	squalene-associated FAD-dependent desaturase	0
blr3002	*hpnD*	farnesyl-diphosphate farnesyltransferase	< 9e^−136^
blr3001	*hpnC*	squalene synthase	< 1e^−131^

^a^The top BlastP hits to each of the Bd110*spc4* genes listed were to species in the following genera: *Tardiphaga*, *Oligotropha*, Afipia, *Nitrobacter* and *Rhodopseudomonas*. The gene numbering is that of strain USDA110. Top BlastP hits to orthologous genes from *Rhodpseudomonas palustris* strain CGA009 were also to species in these genera. The highest expect value for the all the gene homologues identified by BlastP is given. The NCBI RefSeq select protein database was used for the BlastP searches.

## Discussion

In our (MP2) library, which is available to the community as a shared resource, a total of 7347 genes were found to harbor at least one insertion located in the central 5 to 90% of gene length out of the 8094 genes (8317 genes minus the 223 genes deleted). This corresponds to insertions in 90.7% of genes. This Tn library and the accompanying files that document the sites of insertions of T24 should be useful to investigators in several different contexts. One would be to use the Tn library to determine genes important for success in the soybean rhizosphere or in root colonization prior to root nodule formation. A study like this was recently carried out with *Rhizobium leguminosarum* bv. *viciae*^28^*.* Also, since it is quite time consuming to construct *B. diazoefficiens* mutants, inspection of Table S4 can provide confidence that the gene to be disrupted is not essential and that it should be possible to obtain the mutant in question. In addition, a particular gene may be deemed non-essential, but close inspection of the domain structures of the gene may reveal that some domains are likely to be essential. Experimental validation of essential gene candidates by manually deleting genes and checking their phenotypes is always important^29, 30^.

From this analysis we identified a set of five genes that we hypothesize may be essential or conditionally essential for the viability of closely related members of the family *Bradyrhizobiaceae*. The five genes include four genes for synthesis of hopanoid lipids. The *Bradyrhizobiaceae* encompass physiologically disparate bacteria including the nitrate-oxidizing genus *Nitrobacter*, the CO, CO_2_ and H_2_ -oxidizing species *Oligotropha carboxidovorans,* the metabolically versatile phototrophic genus *Rhodopseudomonas*, and the animal-associated bacterium *Afipia felix*, in addition to *Bradyrhizobium.* Despite their diverse physiologies, it seems likely that, unlike other bacteria, most of which do not make hopanoids, this subgroup pf *Bradyrhizobiaceae* rely on membrane hopanoids for full viability in diverse conditions (Table 2).

## Materials and Methods

### Bacterial strains and growth

This study was carried out with *B. diazoefficiens* 110*spc4*, a spontaneous spectinomycin resistant mutant^13^. This strain was a gift from Hauke Hennecke. Bd110*spc4* was grown at 30 °C in arabinose-gluconate (AG) medium^31^.

### Transposon library construction

Transposon mutagenesis was carried out by conjugation of Bd110*spc4* with *E. coli* SM10λpir (pLG107)^11^. Cultures of donor *E. coli* cells [SM10λpir (pLG107)] in mid-exponential growth (OD600 0.2–0.5) were mixed with Bd110*spc4* cells (OD600 0.4–0.8). Cultures (200 ml volumes) were combined in a one-to-one ratio, harvested by centrifugation, suspended in 10 ml phosphate buffer (0.1 M, pH 7) and then filtered onto 10 nitrocellulose discs (0.45 µM pore diameter, Millipore). The filters were placed mating site up on AG plates with no antibiotics. After an overnight incubation, cells from the ten nitrocellulose discs were resuspended in a 1:1 mixture of 0.1 M phosphate buffer and 50% glycerol and stored at -80 °C. The mating suspension was then spread onto 24- by 24 cm QTrays (Molecular Devices) of AG medium supplemented with spectinomycin (100 µg/ml), kanamycin (300 µg/ml) and chloramphenicol (60 µg/ml). A total of 12 matings were carried out. After 10 to 15 days of growth, colonies from each mating were harvested and resuspended in 9 ml of a 1:1 mixture of 0.1 M phosphate buffer and 50% glycerol. One aliquot from each mating was mixed with the others to form master pool 1 (MP1), which was then aliquoted and frozen. To obtain samples for Tn-seq analysis, we thawed two library aliquots, which were treated independently to generate two technical replicates (MP1-A1 and MP1-B1). These two independent samples from MP1 were subjected to high-throughput sequencing. The read values of each technical replicate were normalized to 10^7^ reads to allow comparison between the samples. The total number of reads per insertion location were counted, the genomic locations of the Tn24 insertions were annotated, and read counts per gene were generated for the central 5 to 90% of a gene divided by the full length of the gene or the length of the 5–90% portion of the gene and normalized to reads per kilobase (RpK, or 5–90 RpK_,_ see Table S4). In addition, reads mapping to multiple locations in the genome were counted for each gene. Finally, a combined list of all insertion locations across the two replicates was generated. (Table S1). Technical replicates showed a good correlation of read counts (Figure S1). The columns “MP1-A1 reads” and “MP1- B1 reads” in Table S1 indicate the numbers of multiple and uniquely mapped reads, whereas “MP1-A1 Multi” and “MP1- B1 Multi “columns include the numbers of reads that mapping to multiple locations in the genome. We used the same approach to analyze the MP2 samples (Table S2).

Using our custom bioinformatics pipeline described in Pechter et al.^11^, a total of 68,971 unique insertion location sites were identified and supported by at least two reads. However, 93 to 95% of the reads failed to align to the chromosome of Bd110*spc4*. Instead, most of the reads aligned to the pLG107 plasmid suggesting that the plasmid was retained in Bd110*spc4* after conjugation either as a replicating vector or integrated into its chromosome. To measure the proportion of plasmids relative to inserted transposons, we performed real time PCR using primer sets specific for the transposon and specific for the plasmid backbone on DNA extracted from (i) the MP1 master pool, (ii) the MP1 pool grown in AG plus Km and Spc liquid culture (about 7 generations) and (iii) the MP1 pool re-plated on AG plates supplemented with Km and Spc (real-time PCR conditions and primers are available on request). The values obtained from the real time PCR were analyzed as a ratio of transposon-specific DNA to plasmid backbone-specific DNA. We obtained a ratio of about 1 with DNA extracted from the MP1 pool, confirming the presence of pLG107 in the MP1 cells; about 10 with the liquid grown MP1 cells, and about 150 with the re-plated MP1 pool, suggesting that most of the plasmid was cured from the cells when the MP1 cells were re-plated. Therefore, an aliquot of master pool 1 was thawed and plated onto 24- by 24 cm QTrays (Molecular Devices) of AG medium supplemented with Spc (100 µg/ml) and Km (100 µg/ml). After 15 to 20 days about 360,000 individual colonies were collected in a 1:1 mixture of 0.1 M phosphate buffer and 50% glycerol to create master pool 2 (MP2). The prolonged period of growth was necessary to allow sufficient cell replications (about 30 generations) for the suicide plasmid pLG107 to be lost from cells and for growth of slow growing transconjugants. Master pool 2 was aliquoted (96 tubes of 1 ml aliquots) and frozen at −80 °C. Three aliquots of MP2 (MP2-M1, MP2-H1 and MP2-H2) were subjected to high-throughput sequencing and the genomic locations of the Tn24 insertions were mapped (Table S2). Technical replicates showed a good correlation of read counts (Figure S2). Sequencing of MP2 indicated that about 70% of the reads mapped to the *B. diazoefficiens* genome. The unique transposon insertion sites MP1 and MP2 are listed in Table S3.

### Tn-seq Illumina sequencing

Bd110spc4 Tn library DNA (MP1 and MP2) was prepared for Illumina sequencing using the TdT method, with some modifications^19, 32, 33^. Genomic DNA was extracted using the Qiagen DNeasy kit, eluted in water and quantified using the Qubit kit (Life Technologies). Total genomic DNA used for shearing step for each sample was 2.5 µg with a final volume in each Covaris tube at 130 µl (Covaris E210, 10% duty cycle, intensity of 5, 100 cycles per burst, 200-s duration). Sheared samples were end-repaired using the NEBNext End repair reagents (NEB) following manufacturer instructions. End-repaired DNA was purified (MinElute PCR Cleanup kit) and eluted in EB. For each sample, 1 µg of purified end-repaired DNA was mixed with a 2 µl solution containing 0.5 mM ddCTP (Affymetrix) and 9.5 mM dCTP, 4 µl of 5X TdT reaction buffer, 1 µl of terminal deoxynucleotidyl transferase (Promega) in a final volume of 20 µl. Each sample was incubated 1 h at 37 °C then 20 min at 75 °C and purified using Performa DTR gel filtration columns (Edge Biosystems). To amplify fragments containing the transposon a first qPCR was performed with 5 µl of purified C-tailed DNA as template and primers olj376 (GTGACTGGAGTTCAGACGTGTGCTCTTCCGATCTGGGGGGGGGGGGGGGG) and T24-88 (GATCCCCCTAGGGCGCGCCGAAG) at 250 nM each using SsoFast EvaGreen supermix (Biorad) in 50-µl reactions. The following real time PCR program was used: initial denaturation at 95 °C for 2 min 30 s, and then 24 cycles of 95 °C for 25 s, 59 °C for 25 s, 72 °C for 30 s. A second qPCR was then performed using the same reagents but with 1 µl of the previous PCR product and primers T23_SLXA_PAIR_AMPF_3.

(AATGATACGGCGACCACCGAGATCTACACTAGAGAATAGGAACTTCGGAATAGGAACTTCTTAGATGTGTATAAGAG) and TdT_Index_1_ATCACG.

(CAAGCAGAAGACGGCATACGAGATCGTGATGTGACTGGAGTTCAGACGTGTGCTCTTCCGATCT). The same real time PCR program was used except that 14 cycles were performed. No sample was multiplexed. DNA was purified with the MinElute PCR purification kit (Qiagen) and eluted in 12 µl EB. Samples were quantified using fluorometry (Qubit) and samples quality was checked using the TapeStation system (Agilent). Samples were sequenced with 36-bp single end reads on an Illumina Hi-seq instrument (Genomic Resources at Fred Hutchinson Cancer Research Center) for MP1-A1, MP1-B1, MP2-H1 and MP2-H2 samples and on an Illumina Mi-seq instrument (from the Manoil laboratory, University of Washington) for MP2-M1 sample, using sequencing primer T23_SEQ_G.

(AATAGGAACTTCGGAATAGGAACTTCTTAGATGTGTATAAGAGACAG). Tn-seq sequencing data were analyzed according to the pipeline and protocols described previously^11^.

### Determination of putative essential genes

To determine the set of putative essential genes, the log2 RpK values for Tn insertions in each gene, determined as described above, were plotted and subjected to Gaussian distribution analysis. Essential candidates were selected with log2 5–90 RpK values falling outside a 99.9% confidence interval (P < 0.001 by two-tailed test). The essential candidates were constrained by overlapping the essential candidates of five sequencing runs for the two master pools (MP-1 and MP-2); MP1-A1, MP1-B1, MP2-M1, MP2-H1 and MP2-H2. In addition, genes, particularly short genes, may escape transposition insertion by chance. The probability of escaping insertion can be calculated using the Poisson formula; P(0) = e^-(l)^ = e^(length/58.7)^ where l is the expected value of insertions calculated by the length of the gene divided by the average spacing, 58.7 bp . Based on this, genes of < 310 bp were expected to have no Tn insertions (P-value < 0.005). Thus, these genes were removed from consideration as essential.

### Bioinformatic resources

DEG (database of essential genes, http://tubic.tju.edu.cn/deg/) was used to blast the essential homologues, WebMGA (http://weizhong-lab.ucsd.edu/metagenomic-analysis/) for Cluster of orthologous groups (COG) analysis, CGView Server (http://stothard.afns.ualberta.ca/cgview_server/), for the visualization of putatively essential genes in the context of the *B. diazoefficiens* 110*spc4* chromosome.

### Statistical analyses

Statistical analyses were performed using Excel and R (http://www.R-project.org/).

## Datasets

All data generated or analyzed during this study are included in this published article and its Supplementary Information files.

## Supplementary Information

Supplementary Information 1.

Supplementary Information 2.

Supplementary Information 3.

Supplementary Information 4.

## References

[1] Delamuta JRM (2013). Polyphasic evidence supporting the reclassification of Bradyrhizobium japonicum group Ia strains as Bradyrhizobium diazoefficiens sp. nov. Int. J. Syst. Evol. Microbiol..

[2] Kuykendall L, Elkan G (1976). *Rhizobium japonicum* derivatives differing in nitrogen-fixing efficiency and carbohydrate utilization. Appl. Environ. Microbiol..

[3] Schubert KR, Jennings NT, Evans HJ (1978). Hydrogen reactions of nodulated leguminous plants II. Effects on dry matter accumulation and nitrogen fixation. Plant Physiol..

[4] Itakura M (2009). Genomic comparison of *Bradyrhizobium japonicum* strains with different symbiotic nitrogen-fixing capabilities and other Bradyrhizobiaceae members. ISME J..

[5] Mathis JN, McMillin DE, Champion RA, Hunt PG (1997). Genetic variation in two cultures of *Bradyrhizobium japonicum* 110 differing in their ability to impart drought tolerance to soybean. Curr. Microbiol..

[6] Kaneko T (2002). Complete genomic sequence of nitrogen-fixing symbiotic bacterium *Bradyrhizobium japonicum* USDA110. DNA Res..

[7] Gawronski JD, Wong SM, Giannoukos G, Ward DV, Akerley BJ (2009). Tracking insertion mutants within libraries by deep sequencing and a genome-wide screen for Haemophilus genes required in the lung. Proc. Natl. Acad. Sci..

[8] Langridge, G. C. *et al.* Simultaneous assay of every Salmonella Typhi gene using one million transposon mutants. *Genome Res.* **19**, 2308–2316 (2009). 10.1101/gr.097097.109 PMC2792183 19826075

[9] van Opijnen T, Bodi KL, Camilli A (2009). Tn-seq: high-throughput parallel sequencing for fitness and genetic interaction studies in microorganisms. Nat. Methods.

[10] Goodman AL, Wu M, Gordon JI (2011). Identifying microbial fitness determinants by insertion sequencing using genome-wide transposon mutant libraries. Nat. Protoc..

[11] Pechter KB, Gallagher L, Pyles H, Manoil CS, Harwood CS (2016). Essential genome of the metabolically versatile alphaproteobacterium *Rhodopseudomonas palustris*. J. Bacteriol..

[12] Chao MC, Abel S, Davis BM, Waldor MK (2016). The design and analysis of transposon insertion sequencing experiments. Nat. Rev. Microbiol..

[13] Regensburger B, Hennecke H (1983). RNA polymerase from *Rhizobium japonicum*. Arch. Microbiol..

[14] Fernández N (2019). An integrated systems approach unveils new aspects of microxia-mediated regulation in *Bradyrhizobium diazoefficiens*. Front. Microbiol..

[15] Dembek M (2015). High-throughput analysis of gene essentiality and sporulation in *Clostridium difficile*. MBio.

[16] Gislason AS, Turner K, Domaratzki M, Cardona ST (2017). Comparative analysis of the Burkholderia cenocepacia K56–2 essential genome reveals cell envelope functions that are uniquely required for survival in species of the genus Burkholderia. Microbial Genom..

[17] Yang H (2014). Genome-scale metabolic network validation of *Shewanella oneidensis* using transposon insertion frequency analysis. PLoS Comput. Biol..

[18] Christen B (2011). The essential genome of a bacterium. Mol. Syst. Biol..

[19] Klein BA (2012). Identification of essential genes of the periodontal pathogen *Porphyromonas gingivalis*. BMC Genom..

[20] Wu S, Zhu Z, Fu L, Niu B, Li W (2011). WebMGA: a customizable web server for fast metagenomic sequence analysis. BMC Genom..

[21] Green B, Bouchier C, Fairhead C (2012). Insertion site preference of Mu, Tn5, and Tn7 transposons. Mob. DNA.

[22] Martinez-Romero E, Ormeño-Orrillo E (2019). A genomotaxonomy view of the *Bradyrhizobium* genus. Front. Microbiol..

[23] Belin BJ (2018). Hopanoid lipids: from membranes to plant–bacteria interactions. Nat. Rev. Microbiol..

[24] Silipo A (2014). Covalently linked hopanoid-lipid A improves outer-membrane resistance of a Bradyrhizobium symbiont of legumes. Nat. Commun..

[25] Furubayashi M, Li L, Katabami A, Saito K, Umeno D (2014). Construction of carotenoid biosynthetic pathways using squalene synthase. FEBS Lett..

[26] Perzl M (1998). Cloning of conserved genes from Zymomonas mobilis and *Bradyrhizobium japonicum* that function in the biosynthesis of hopanoid lipids. Biochimica et Biophysica (BBA) Acta Lipids and Lipid Metabolism.

[27] Kulkarni G (2015). Specific hopanoid classes differentially affect free-living and symbiotic states of *Bradyrhizobium diazoefficiens*. MBio.

[28] Wheatley RM, Ford BL, Li L, Aroney STN, Knights HE (2020). Lifestyle adaptations of *Rhizobium* from rhizosphere to symbiosis. Proc Natl Acad Sci U SA..

[29] Rubin BE (2015). The essential gene set of a photosynthetic organism. Proc. Natl. Acad. Sci..

[30] Lee SA (2015). General and condition-specific essential functions of *Pseudomonas aeruginosa*. Proc. Natl. Acad. Sci..

[31] Sadowsky MJ, Tully RE, Cregan PB, Keyser HH (1987). Genetic diversity in *Bradyrhizobium japonicum* serogroup 123 and its relation to genotype-specific nodulation of soybean. Appl. Environ. Microbiol..

[32] Gallagher, L. A. *et al.* Resources for genetic and genomic analysis of emerging pathogen *Acinetobacter baumannii*. *J. Bacteriol.* **197**, 2027–2035 (2015). 10.1128/JB.00131-15 PMC4438207 25845845

[33] Lazinski DW, Camilli A (2013). Homopolymer tail-mediated ligation PCR: a streamlined and highly efficient method for DNA cloning and library construction. Biotechniques.

