# Supplementary material for: Transposon sequencing analysis of Bradyrhizobium diazoefficiens 110spc4
Source: Sci Rep. 2021 Jun 24;11:13211. doi: 10.1038/s41598-021-92534-z (PMC8225791; doi:10.1038/s41598-021-92534-z)
Supplement: Supplementary file 1 — Supplementary Information 1. [file 41598_2021_92534_MOESM1_ESM.docx]

**Supplemental Information**

**Transposon sequencing analysis of *Bradyrhizobium diazoefficiens* 110*spc4***

Claudine Baraquet, Weijun Dai, Jose Mendiola, Kieran Pechter and Caroline S. Harwood

**Figure S1**. Correlations of Tn-seq sample replicates. Two independent technical replicates of the Bd110*spc4* T24 library genomic DNA were extracted from cells and prepared for high-throughput sequencing. The read values for each gene were determined for both replicates and plotted to determine the correlation.


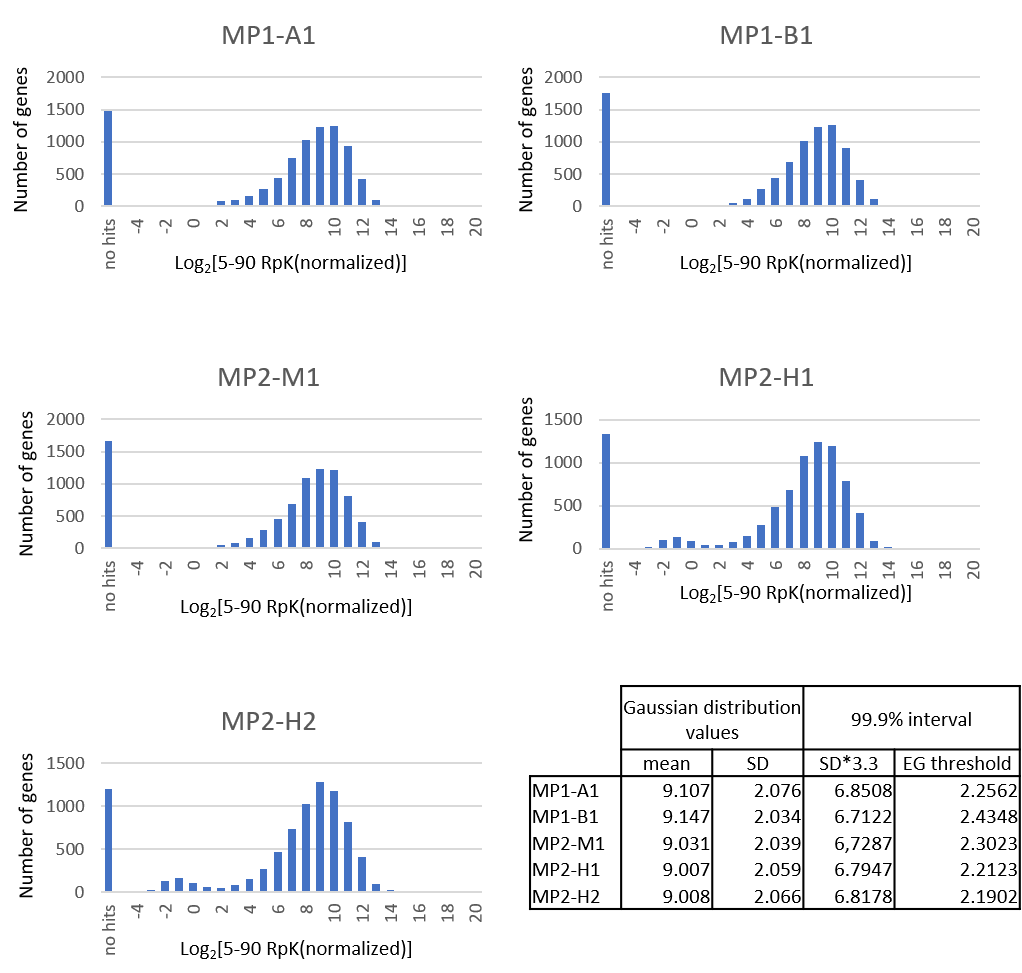


**Figure S2.** Distribution of RpK values among Bd110*spc4* genes. Read counts of each sample sequenced were normalized to 10^7^ reads, gene length and tallied for the 5 to 90% length window of each gene to give reads-per-kilobase (RpK) values. Log_2_[5-90 RpK(normalized)] values were calculated, and histograms were generated. Histograms were generated for each sample (as indicated at the top of the graph). The number of genes with no hits and no reads were also indicated (log_2_ of zero is undefined). Log_2_[5-90 RpK(normalized)] were then fit to a normal distribution using the non-linear least squares fit and the means and standard deviations of the distribution were calculated and used to determine the 99.9% confidence intervals. The values of mean and standard deviation of each distribution are indicated in the Table. Genes with Log_2_[5-90 RpK(normalized)] values below the EG threshold value (mean minus 3.3 SD for the 99.9% interval) were considered as putatively essential.

**Supplementary data files**

**Table S1**. MP1 - Tn-seq normalized read values per insertion for Bd110*spc*4 using strain USDA110 as the reference genome.

Reads from the two samples (MP1-A1 ad MP1- B1) of the MP1 library were mapped to strain USDA110 as the reference strain. The position and the direction (reverse or forward) of each Tn insertion as well as information on the gene where the insertion occurred (locus tag, start and stop codon, strand, gene name, protein product) are indicated. The position of the transposon insertion relative to the start site is also indicated (the number written in brackets indicates the gene length). The number of reads observed for each position of a Tn insertion in samples MP1-A1 and MP1-B1 corresponds to the unique and multiple mapped reads (column MP1-A1 Reads and MP1-B1 Reads). The read values of each technical replicate were normalized to 10^7^ reads. These values were taken into consideration for the analysis. MP1-A1 Multi and MP1- B1 Multi indicate the multiple mapped reads.

To access data go to: <https://figshare.com/account/home#/data>.

Log into: [csh5@uw.edu](mailto:csh5@uw.edu) / Brady110spc (password)

**Table S2**. MP2 - Tn-seq normalized read values per insertion for Bd110*spc*4 using strain USDA110 as the reference genome.

Reads from three samples (MP2-M1, MP2-H1 and MP2-H2) of the MP2 library of Bd110*spc4* were mapped to strain USDA110 as the reference strain. The position and the direction (reverse or forward) of each Tn as well as information of the gene where the insertion occurred (locus tag, start and stop codon, strand, gene name, protein product) are indicated. The position of the transposon insertion relative to the start site is also indicated (the number written in brackets indicates the gene length). The number of reads observed for each position of a Tn insertion in samples MP2-M1, MP2-H1 and MP2-H2 corresponds to the unique and multiple mapped reads (column MP2-M1 Reads, MP2-H1 Reads and MP2-H2 Reads). The read values of each technical replicate were normalized to 10^7^ reads. These values were taken into consideration for the analysis. MP2-M1 Multi, MP2-H1 Multi and MP2-H2 Multi indicate the multiple mapped reads.

To access data go to: <https://figshare.com/account/home#/data>**.**

Log into: [csh5@uw.edu](mailto:csh5@uw.edu) **/** Brady110spc (password)

**Table S3.** Unique transposon insertion sites in Bd110*spc*4 mapped to the USDA110 genome. The Table combines the transposon insertions from both the MP1 and MP2 libraries leading to 155,042 unique Tn insertions.

To access data go to**:** [**https://figshare.com/account/home#/data**](https://figshare.com/account/home#/data)**.**

Log into: [csh5@uw.edu](mailto:csh5@uw.edu) / Brady110spc (password)

**Table S4**. Tn-seq RpK values for all Bd110*spc4* genes using strain USDA110 as the reference strain.

The number of insertions (Hits) and reads (RpK) was counted for each gene (entire gene length or the central 5 to 90% of gene length). Reads count per gene were normalized to gene length (1 kb = 1 gene), leading to normalized reads per kilobase (RpK) values for each gene. The log_2_ 5-90 RpK for each gene was then calculated. The designation "-" is used when RpK=0 because log_2_ of zero is undefined. Z-scores were calculated from the following formula. z = (X - μ) / σ where X is the log2RpK value, μ is the population mean, and σ is the standard deviation. Highlighted values are z scores <-3.3 (red, potential essential genes, 99,9% interval), or z-scores of between -3,3 and -1.96 (orange, reduced fitness genes, 95% interval). p-value = e^(-gene lenght/F)^ where F=genome size/number of unique insertions. Number of unique insertions is based on the total number of unique insertions for the five experiments (MP1-A1, MP1-B1, MP2-M1, MP2-H1 and MP2-H2; 155,042 unique Tn insertions). Highlighted values in green have a p-value <0.005.

**Table S5**. Bd110*spc4* putative essential gene set. The 223 genes deleted in Bd110*spc4* were removed from the list, leading to 622 putative essential genes. Genes highlighted in yellow belong to the symbiosis island.

**Table S6**. COG functional assignments of Bd110*spc4* putative essential genes.
